# COVID-19 pandemic: impacts on the achievements of Sustainable Development Goals in Africa

**DOI:** 10.11604/pamj.2021.38.251.27065

**Published:** 2021-03-11

**Authors:** Goodness Ogeyi Odey, Abrar Gamal Abdallah Alawad, Ouma Sarah Atieno, Elsa Olubunmi Carew-Bayoh, Esther Fatuma, Isaac Olushola Ogunkola, Don Eliseo Lucero-Prisno III

**Affiliations:** 1 Department of Public Health, University of Calabar, Calabar, Nigeria,; 2 School of Pharmacy, Ahfad University for Women, Khartoum, Sudan,; 3 School of Medicine, Maseno University, Kisumu, Kenya,; 4 Department of Surgery, University of Sierra Leone/Teaching Complex Hospital, Connaught Hospital, Freetown, Sierra Leone,; 5 Research Department, University of Goma, Goma, Democratique Republic of Congo,; 6 Department of Global Health and Development, London School of Hygiene and Tropical Medicine, London, United Kingdom

**Keywords:** Africa, Sustainable Development Goals, COVID-19

## Abstract

Since the launch of the Sustainable Development Goals (SDGs) in 2015, Africa has made some progress towards its achievements, particularly in the area of gender equality- SDG 5, climate change -SDG 13 and preserving life on land- SDG 15. Although, some African countries have made tangible impact on the other goals such as health, food production and economic growth; these efforts experienced some setbacks with attention shifting to curbing the spread of the novel coronavirus. As much as mitigating the spread of the spread of COVID-19 is important, so is ensuring efforts made on other goals are not lost, as the SDGs are much interconnected, and failure in one impacts others. For the African continent to achieve sustainable development beyond COVID-19, strategic actions which will involve innovations, evaluations and strong political will towards implementations must be taken by relevant stakeholders, so the continent is not left behind in the global goals achievement by 2030.

## Commentary

The Sustainable Development Goals (SDGs) also known as Global Goals, are 17 strategic goals that were developed and adopted by all United Nations member states in 2015 as a universal call to action for the eradication of poverty, protection of the planet, and ensuring that all peoples enjoy peace and prosperity by 2030. These SDGs were integrated recognizing that action in one area would affect outcomes in others and that development must balance socio-economic, and environmental sustainability [1]. Since their inception, the pledge of the Sustainable Development Goals was to leave no one behind. The SDGs were a roadmap and a commitment by the world to ensure optimum human and planetary health and prosperity by 2030, starting with those at the furthest back [2]. Different countries have made varied progress on the 17 global goals, including Africa, which has made slow but tangible steps towards achieving this developmental agenda, until 2020 [1, 2].

The COVID-19 pandemic hit the world on an unprecedented scale, bringing all aspects of the world to a stand-still and regression at a pace never before experienced, including, and most especially, developmental agendas. In what has been described as a twin crisis - an economic and health crisis [3], the world is grappling with new and pre-existing challenges all around. Although Africa lags behind on the COVID-19 pandemic curve, accounting for 1.5% of cases confirmed globally as of 9 May 2020, the region is set, by way of the health and economic crises, to lose traction in the achievement of the 2030 goals [3]. Government and health systems are bombarded with testing, managing, and monitoring COVID-19, while the economy grapples with the fiscal implications of all these responses and activities, in a way that if done shortsightedly, could cause the world to lose focus of the future goals of the Sustainable Development agenda. Data releases by SDG indexes and trackers worldwide, including the Sustainable Development Report (June 2020) [2], highlight that clearly, the pandemic will have profound implications on progress towards the SDGs worldwide, including those of Africa.

### The state of SDGs in Africa

Globally, the world has been on track to end poverty (SDG1), making a steady decline from 15.7% in 2010 to 8.2% in 2019 with projections of reducing to 6% by 2030. SDG2 (food insecurity) had already been on the rise from 22.4% in 2014 to 25.9% in 2019. The improvement had also been made on SDG 3 (health) but there is a great need for acceleration [2]. According to the International Monetary Fund (IMF) and the World Bank, Africa prior to COVID-19 had five of the fastest-growing economies globally; Ghana, South Sudan, Rwanda, Ethiopia, and Côte d´Ivoire. In 2019, Ghana led the pack with a projected expanding economy globally at 8.79%. This growth was due to macroeconomic gains like single-digit inflation, fiscal consolidation, and a clean-up of the banking sector. Overall, the African economic growth was forecast to pick up to 3.9% in 2020 and 4.1 percent in 2021, in spite of the continent economic giants-Nigeria and South Africa-growth slowing down [4]. However, this current positive trajectory is juxtaposed by weak governance systems, poor leadership, corruption, crippling high debt, and fragile and conflict-affected situations witnessed in 20 countries in Africa [5].

Despite the widespread adoption of and progress toward the Sustainable Development Goals, Africa continues to lag behind most of the world when it comes to socio-economic development. As a matter of fact, a report by the Sustainable Development Goals Center for Africa- Africa 2030: Sustainable Development Goals Three-Year Reality Check, reveals that minimal progress has been made and, in some instances, there was a complete stagnation towards SDG targets [5]. With approximately 10 years to go, Africa has only shown progress towards meeting three out of the seventeen goals: SDG 5 (gender equality), SDG 13 (climate action), and SDG 15 (Life on land) [5]. Prior to COVID-19, the growth recorded for the region over the SDG period was below the SDG target of 7% per year and was at 1.4% in 2016 [5]. Concentrated poverty remains high and inequality in Africa ranks amongst the highest in the world. More than half of the global poor (those who earn under $1.90 PPP per day) are found in Africa with one in every three Africans at risk of food insecurity. Education systems in continent lag significantly behind as compared to other regions within the world and no African nation has attained universal primary education: 63.3 million primary age kids are out of school, of which 34.1 million (54%) are found in sub-Saharan Africa and 56% of them are girls [5].

Furthermore, health indicators in the continent are also low compared to alternative regions of the planet with an outsized funding chasm required to be filled to achieve SDG3. The under-five and neonatal death rate in 2016 were eight and five times higher in the region than that in Europe respectively and the maternal mortality rate as of 2015 in the continent was at 542 per 100,000 live birth [5]. In Africa, there are about 12.8 skilled health employees per 10,000 population in Africa, compared to 115.3 per 10,000 in Europe. Even though Africa suffers greater than 24% of the global burden of disease, it contributes only 3% of the world´s health workers and less than a percentage of the world´s financial resources [5]. Although some areas of the region have shown some promises regarding the determination of governments in establishing action plans for implementation of the SDGs and also assigning specific institutions to lead the work on data and indicators, there is a need for serious actions towards achieving the SDGs.

### Challenges posed by COVID-19 on SDGs

COVID-19 has been a systemic crisis in human development, but this pandemic became unprecedented because it is an evolution from health shock to an economic and social crisis. This pandemic has had great challenges to all countries and affects SDGs achievement [1, 6]. As of 1^st^, September 2020, Africa Centre for Disease Control and Prevention (CDC) brief reported that the Southern Africa region has the highest confirmed cases 35% (20,146) followed by the Northern region 29% (16,691), Eastern region 24% (13,911), Western region 9% (4,923) and Central region 3% (1,600) [7]. The pandemic suppressed the routine work of vaccination and surveillance of vaccine-preventable diseases in Africa and led to outbreaks of vaccine-preventable diseases like measles and malaria in Chad, South Sudan, and Ethiopia [3]. The International Food Policy Research Institute (IFPRI) 2020 report predicted that Africa could face 64.9 million malnourished patients in addition to 282 million already malnourished patients existing in 2020 [8].

Lockdowns limited the access to sexual and reproductive health services and reduced women contact with Health facilities, according to United Nations Fund for Population and Activities (UNFPA) 2020 report, it predicted that Africa will suffer from 253,500 additional child deaths and 12,200 additional maternal deaths, and there is a risk of reversing of HIV/AIDS according to WHO report [3]. The economic impact of the COVID-19 pandemic in Africa countries has been tremendous, with depletion of domestic public resources by affecting tax and non- tax revenues, due to lockdowns and travel restriction there is a significant decline in tourism-which accounts for about 20% of the Gross National Product of some countries, international trade and suppression of domestic consumption revenue which most of Africa countries rely on. Similarly, 85.8% of the workforce in informal sector workers lost their jobs due to lockdown, depreciation of the local currency, and deterioration in the current account balance [9]. According to World Bank Development Indicators, this pandemic will maintain a poverty unfinished agenda and move Africa further off track the SDGs target, with an additional 81,928,000 people living in poverty due to COVID-19 [3].

Due to travel and export restrictions on rice and wheat, this has led to food insecurity in Africa with a sharp rise in food prices and rising hunger and malnutrition [9]. Reports from Food and Agriculture Organization (FAO) indicated that 21.5% of 1,288 million Africans were suffering from severe food insecurity. Similarly, the World Food Programme (WFP) 2020 report suggests that more than 73 million people from African countries will experience food crises and food insecurity [3]. Prolonged school closure in Africa left over 330 million learners of all levels and over 8.5 million teachers, unable to learn or teach from home, around 56 million live in areas not served by mobile networks, 90% of students have no access to home computers and 82% are not able to get online. Unreliable power supply and poor internet connection, coupled with financial costs are some of the reasons that affect educational achievement in Africa [8].

According to the UNFPA report, there is an increase in sexual and gender violence, affecting children, including in refugee and internally displaced persons (IDP) camps and conflict-affected areas. In Nigeria, there was a sudden upsurge of protests and crimes in the country during the pandemic [10]. Stigmatization and xenophobia against positive COVID-19 patients, foreigners, refugees, migrants, and nationals returning to their home countries has been noted in some regions [8]. The lockdown during the pandemic, international restrictions, poor connectivity issues, and deterioration in the economic situation led to staff reduction, limitation of missions and supplies, and an increase in humanitarian needs in Cameroon, Burundi, and Chad [9]. Even with recovery measures, the effect of the pandemic on the continent is seen in the negative growth in most sectors, especially the progress of the SDGs. Studies have shown that more people have been pushed into poverty- half a billion Africans [1], hunger, malnourishment and weakened food supply chains, loss of decent work- 1 in 6 young people lost their jobs with COVID-19, breakdowns in education that re-emphasize inequalities like gender and the digital divide, with 1.25 billion African students out of school, reduced access to healthcare, slowed infrastructural growth, interrupted government and democratic functions like elections and referendums, which provide a breeding ground for human rights violations [1].

Due to the largesse and complexity of the COVID-19 outbreak, Africa´s authorities may be distracted and overwhelmed, with negative implications for the focus on futuristic developmental agendas. The general consensus is that with COVID-19, the continent´s current strategic trajectory arguably stifles effective implementation of the SDGs, and poses risks to the realization of 2030 targets [3]. It is worth noting that Asia-Pacific countries that ranked the highest in SDG scores also ranked the highest in the handling of COVID-19 [2]. It can thus be seen that focusing on the goals would be beneficial for any country in the present and likely future pandemics. Besides dealing with COVID-19, Africa needs to stay the course on the SDG agenda, for the good of its people.

As momentum geared towards the achievement of the global goals especially in this decade of action gradually picks up amidst the storms that came with the pandemic, in as much as focusing on the mitigation of the virus is important, so is the future of the planet and the people of the African continent. If no one is to be left behind, as pledged, then there is a need for creative, futuristic thinking that encompasses the emerging practices and complexities that evolved in the midst of the pandemic, resilience, and innovations in policy designs and implementation. Concerted efforts are needed, both African and internationally, to ensure that the gains made by the continent for SDGs are not undone by the COVID-19 pandemic and that the continent continues to achieve developmental progress.
